# Data-driven modeling for bond strength in reinforced concrete structures

**DOI:** 10.1038/s41598-026-45119-7

**Published:** 2026-04-10

**Authors:** Priyanka Singh, Sandeep Singla, Aarti Bansal, Manish Kumar, Akanksha Mrinali, S. M. Mozammil Hasnain

**Affiliations:** 1 Department of Civil Engineering, Amity School of Engineering and Technology, Amity University, Noida, Uttar Pradesh India; 2 Chitkara University Institute of Engineering and Technology, Chitkara University, Patiala, Punjab India; 3 Department of Electronics and Communication Engineering, Thapar Institute of Engineering and Technology, Patiala, Punjab India; 4 Department of Civil Engineering, GD Goenka University, Sohna Rural, India; 5 Department of Artificial Intelligence and Machine Learning, Manipal University Jaipur, Jaipur, 303007 Rajasthan India; 6 Department of Mechanical Engineering, Faculty of Engineering and Technology, Marwadi University Research Center, Marwadi University, Rajkot, 360003 Gujarat India

**Keywords:** Bond strength, Reinforced concrete, High temperature, Machine learning, Prediction, Engineering, Materials science, Mathematics and computing

## Abstract

In reinforced concrete (RC) structures bond strength is critical mechanical parameters responsible for the load transfer, cracking behavior, and structural integrity, especially for higher temperature scenarios. Research framed, data driven, data reinforced predictive model is constructed to estimate the normalized bond strength of reinforced concrete under high-temperature scenarios using the techniques of machine learning. A total of 458 data points from various publications were assembled, and for the purpose of training and evaluating the machine learning regression models, the ensemble and the kernel-based and neural network techniques, several machine learning algorithms were utilized. A modeling protocol ensuring robust generalization with standardization, repeated cross validation, and data driven hyper parameter optimization was used for the purpose of comparative modeling. The XGBoost regressor was the most predictive among the 12 regression models created, with a total coefficient of determination of R2 = 0.9417 with a root quadratic mean error (RQME) of 7.9 on the testing data set. Further analysis of important predictive factors, such as degradation of bond strength, and the exposure temperature, provide credible, data driven and rational emphasis for the geometric parameters of the predictive model. The suggested framework illustrates that, for estimating the bond strength of reinforced concrete exposed to high-temperatures, machine learning is not only an effective tool, but also one that provides the assessments in a timely manner. Thus, it can be used in the areas of structural fire assessment, post-fire evaluation, and durability design.

## Introduction

The reinforcement and concrete bonding properties own together define compatibility and performance. The bond strength is one of the influential mechanical properties of RC structures^1^. Concrete bond strength prediction holds great value in the domain of structural engineering and construction^2^. Bond strength is defined as concrete’s ability to create bonds, typically with reinforcing steel rods (rebar) or pre-stressing tendons, and is one of the critical attributes for the structural integrity and functioning of RC structures. There are considerable challenges to overcome for an accurate prediction of bond strength, such as (a) there is the issue of Concrete heterogeneity, and an example of its varying properties is with respect to concrete mixing, curing, and the quality of constituent materials used^3,4^. These variations, in turn, cause bond strength prediction to suffer. (b) Concrete’s age and the curing methods applied form an additional variable for bond strength, as older, well-cured concrete can exhibit different bond strength properties than newly placed concrete^5^. (c) The bond strength can change in concrete under different stress states and loading histories.

Predicting changes to bond strength during dynamic or cyclic loading conditions remains to be seen^6^. (d) The bond strength may be affected by the interface geometry and surface roughness between the concrete and reinforcing steel. The impact from the interface geometry and surface roughness is complex to model and predict^7^. (e) Although many guidelines and models exist to predict bond strength, there are still many unaccounted complex characteristics that bond strength models do not address^8^. (f) There is little data concerning the longitudinal performance of concrete structures related to bond strength. There is a data deficiency related to the longitudinal performance of concrete structures that makes predicting the bond strength and durability of concrete extremely complicated.

The application of ML has been employed to assist concrete bond strength predicting setbacks. While ML has been creating pathways to predicting bond strength, due to its data driven approaches and ability to untangle complex relationships in data sets, has help predicting bond strength in concrete^9^. Some of the major contributions of ML in predicting bond strength include the following: (a) compared to typical analytical models, ML predicts bond strength more accurately, due to its ability to identify complex patterns and non-linear relationships within the provided data sets. This is due to the fact that machine learning models consider relationships of multiple variables (material attributes, environmental, surface, and structural) that factor into a configurational drive to bond strength^10–13^. (b) MA models learn from large data sets which helps in adjusting to the variability of the material attributes and its surrounding condition. This in turn helps in predicting reliably in varying data sources^14^. ML creates predictive models using large data sets on concrete attributes, test results, and performance data from the past.

The integration of these datasets could increase the accuracy of their predictions of bond strength^15^. d) ML methods have been applied, as non-invasive methods, to assess the bond strength of concrete structures without the risk of damaging the concrete^16^. e) ML models can identify potential issues with bond strength before they become critical and, thus, provide opportunities for the preventative repair and maintenance of concrete structures^17–21^.

In addition to the mechanical stresses, the use of the structure will introduce other factors that will affect bond-slip characteristics between the steel and concrete. These factors will also be influenced by chemical and physical environments^22,23^. Therefore, it is imperative to study the pattern of variation and bond strength of RC structures^23^. When it comes to bond strength in reinforced concrete (RC) structures, there has been quite a bit of research. Pull-out tests and beam tests^25–28^ are frequently used to study the bond-slip behavior between the reinforcement and the concrete in RC structures. Early researchers used to write down numerous hypothesized equations expressing the bond stress and bond slip, based on the experiments and some theory^29,30^. These models can and should be improved; because of the incomplete factors, models can be influenced in terms of design, systems, and the external environment. This has been the impetus for researchers analyzing the conditions of the loads^31–33^, the stress^34,35^, the shapes and sizes of the reinforcement and concrete^36^. Large proportions of research have been performed on the bond-slip characteristics for conditions of extremes high temperatures^37^, humidity^38^, and corrosion^39^. Thus, multiple factors have been studied, creating numerous models to predict bond-slip behavior more accurately.

Nevertheless, the existing models are still open to refinement in some aspects. These models were constructed and adjusted based on certain empirical datasets and/ or basic principles. At the same time, the models take into account only limited variables and ignore the influence of other factors. It is challenging to obtain dependable predictions and implement the models examined in this context in practical engineering^40^. Recently, machine learning (ML) has been implemented in numerous domains of civil engineering to solve complex problems^41,42^. Studies have also evaluated hybrid optimization strategies, sustainability-driven modeling frameworks, and ensemble learning enhancements to anticipate the performance of structural materials under environmental variability^43,44^.

ML frequently achieves high accuracy and low error by directly exploiting the intrinsic connection between inputs and outputs without requiring intricate mechanical analysis^45^. Simultaneously, an increasing number of ML applications are being utilized to evaluate the binding strength of RC structural interfaces^46^. An approach based on machine learning was suggested to determine the bond strength at RC interfaces^45^. ML method was effectively implemented to forecast the bond strength of concrete structures^48^. In order to generate predictions, researchers have analysed numerous databases through the accumulation of vast quantities of data. A comparison of representative machine learning studies on bond strength prediction is summarized in Table 1. Recent studies have further expanded the application of ensemble and boosting-based learning techniques for predicting bond-related and mechanical properties of concrete materials. ANN and SVR approaches were applied for bond strength estimation under ambient conditions^49^, while Random Forest and XGBoost models were explored for concrete mechanical property prediction^50^. XGBoost was further implemented for bond-related strength modeling^51^. However, these investigations did not explicitly address bond degradation under elevated temperature exposure or incorporate systematic repeated cross-validation combined with interpretability analysis. These limitations motivate the present study. Furthermore, recent works have incorporated hybrid optimization and metaheuristic-assisted regression frameworks to enhance model generalization in civil engineering applications^52,53^. Although these studies demonstrate promising predictive improvements, they do not focus on bond degradation under elevated temperature exposure.

**Table 1:** Comparison of recent machine learning studies on bond strength prediction.

Study	Year	Dataset size	Output variable	Temperature considered	ML models	Best model	Interpretability	Main limitation
Golafshani et al.^7^	2012	156	Bond strength	No	ANN, Fuzzy	ANN	No	Small dataset, ambient only
Su et al.^13^	2021	270	FRP–concrete bond	No	GP, ANN	ANN	No	No thermal effects
Fan et al.^3^	2023	320	Bond strength	No	GWO–SVR	SVR	No	Ambient conditions only
Alyousef et al.^11^	2023	300+	Bond-related properties	Yes (limited)	AdaBoost	AdaBoost	No	Not focused on bond degradation
Zhang et al.^49^	2024	132	Bond strength	No	ANN, SVR	ANN	No	Limited variables
Li et al.^50^	2024	185	Bond strength	No	RF, XGBoost	RF	No	No thermal effects
Chen et al.^51^	2025	160	Bond-related strength	No	XGBoost	XGBoost	No	No interpretability
This study	2026	459	Normalized bond strength	Yes (20–825 °C)	12 ML models	XGBoost	Yes	Comprehensive & interpretable

Despite the significant progress achieved in experimental and data-driven modeling of bond strength, several important research gaps remain. Most existing machine learning studies are developed using limited datasets and focus primarily on ambient temperature conditions, without explicitly addressing the degradation of bond behavior under elevated temperature or fire exposure. Moreover, the majority of available models consider only a small number of influencing parameters and evaluate a restricted set of algorithms, which limits the generalization capability of the proposed approaches. In addition, model interpretability is rarely investigated, and the relative influence of temperature, geometric parameters, and material age on bond degradation is often not quantified. As a result, there is still a lack of a comprehensive, systematically validated, and interpretable prediction framework for bond strength of reinforced concrete under high-temperature conditions.

Although significant progress has been achieved in data-driven bond strength modeling, no previous study has simultaneously (i) constructed a large high-temperature bond degradation database, (ii) conducted a systematic cross-paradigm comparison of twelve regression algorithms under elevated temperature exposure, and (iii) incorporated interpretability analysis to connect data-driven findings with physical bond degradation mechanisms. Existing works typically evaluate a limited number of algorithms under ambient conditions without repeated cross-validation or stability assessment. The present study addresses these combined gaps by providing a rigorously validated and interpretable predictive framework specifically tailored to bond degradation under thermal exposure.

The major contributions of this research can be summarized as follows:

- A comprehensive database consisting of 458 experimental bond strength data points collected from published literature is systematically assembled, with particular emphasis on the degradation of steel–concrete bond under elevated temperature conditions.
- A systematic comparative evaluation of twelve machine learning regression models is conducted to identify suitable predictive frameworks for normalized bond strength, covering a wide spectrum of ensemble, kernel-based, and neural network algorithms.
- A stable and generalizable model is built from a fair comparison of the results after repeated cross-validation, hyperparameter tuning and standardization of the data.
- Among the various error metrics used to measure the predictive accuracy of each suggested model, the XGBoost regressor stands out as the benchmark model for predicting bond strength under extreme heat conditions for its superior accuracy.
- With the help of the data-driven results and the existing mechanical behavior principles of bonds, and the importance of each feature with regard to the influence of temperature, bond strength, and age of the other materials and geometric dimensions, an analysis of interpretability is performed.
- In the fields of structural fire analysis, post-fire evaluation, and design for durability, the proposed framework serves as an effective and pragmatic predictive tool for estimating the bond strength of reinforced concrete at elevated temperatures.

The structure of the paper is as follows: In Sect.  2 we elaborate on the data used for this research and in Sect.  3 we elaborate on the methodology we proposed. Section  4 contains the results of the experiments and the analysis, and the discussion is extensive. Finally, we end with Sect.  5 which contains our conclusions and outlines the potential of the findings.

## Dataset description

### Data source and variable definition

The study’s dataset has 458 experimental records collected from previous studies of pull-out tests published in the literature. Each sample relates to one experimental observation of the bond behavior of reinforced concrete at high temperatures. Only records with fully known geometry, material, and thermal parameter information were kept in the final dataset.

The input variables considered in this study are: (i) fibre type, (ii) fibre volume fraction, (iii) length-to-diameter ratio of the embedded bar (l/d), (iv) cover-to-diameter ratio (c/d), (v) age at testing (days), (vi) thermal saturation parameter $\:d=h/{d}_{m}^{2}$, and (vii) exposure temperature (°C). The output variable is the normalized bond strength (NBS), defined as the ratio of the peak bond strength at elevated temperature to the peak bond strength at ambient temperature.

These variables were selected based on their established influence on bond degradation mechanisms, including thermal damage, confinement effects, embedment geometry, and concrete maturity.

### Descriptive statistics

For a quantitative perspective, Table 2 gives a summary of the descriptive statistics of all inputs and outputs, including the minimum, maximum, mean, std, and interquartile ranges.

Coverage of different experimental conditions is shown in Table 2, where exposure temperatures varied between 20 °C and 825 °C, and bond strength values were between 0 and 114% (in normalized values). Enough variability is given for the solid testing and training of machine learning models for the other parameters, as well as the geometric factors (l/d and c/d), age at testing, and all other intervals quite sufficiently.

**Table 2:** Descriptive statistics of input and output variables (*N* = 458).

Variable	Min	Mean	Std	Median	Q1	Q3	Max
Fibre type	0	0.336	0.849	0	0	0	3
Volume fraction (%)	0	0.126	0.418	0	0	0	2
Length/diameter (l/d)	2.0	9.62	5.75	7.5	5.0	12.5	20.83
Cover/diameter (c/d)	1.78	4.69	1.22	4.85	4.19	5.75	5.75
Age (days)	28	48.0	32.1	28	28	60	150
d (h/dm^2^)	0.333	1.72	1.02	1.39	0.80	3.00	3.00
Temperature (°C)	20	348.6	251.5	350	100	600	825
NBS (%)	0	69.89	28.95	77.72	47.27	97.87	114.24

### Exploratory data analysis

**Fig. 1:**
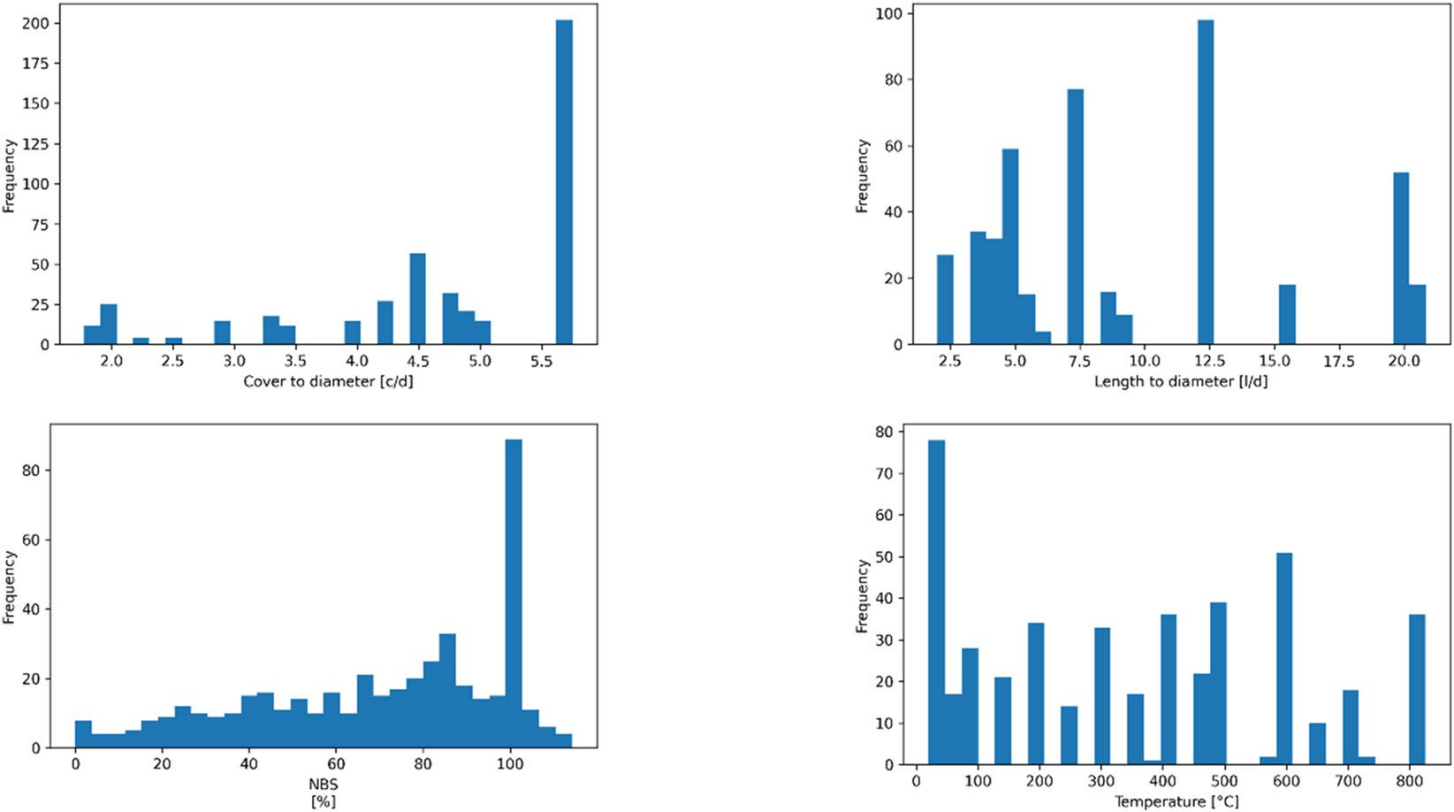
Histograms of key input and output variables.

Model building was preceded by exploratory data analysis (EDA) to determine the starting dataset. Figure 1 shows the histograms for the datasets for the length-to-diameter ratio, cover-to-diameter ratio, exposure temperature, and normalized bond strength. These distributions show variability for the geometric and thermal datasets confirming the dataset are ideal for learning complex and nonlinear relationships.

**Fig. 2:**
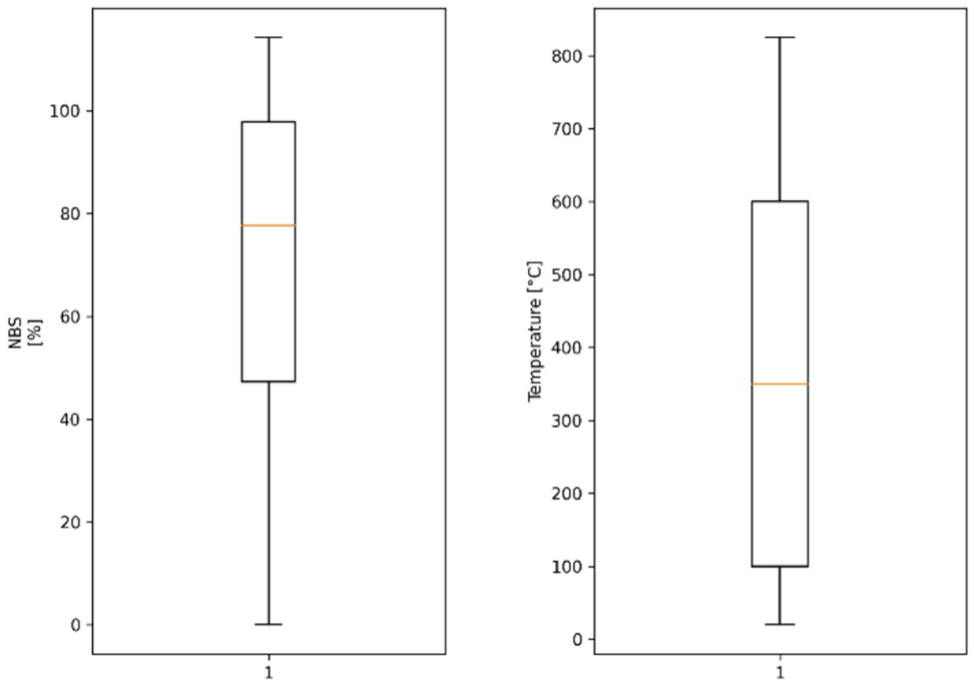
Box plots of exposure temperature and normalized bond strength.

Furthermore, Fig. 2 shows the exposure temperature and normalized bond strength box plots. The moderate box plot suggests and the small extreme outlier plot suggests no severe absent of the value. Thus, no extreme outlier deletions were done and all data points were kept for modeling.

Exploratory analysis indicate the dataset have a wide range of temperature and bond degradation levels which is ideal for modeling.

### Correlation analysis

**Fig. 3:**
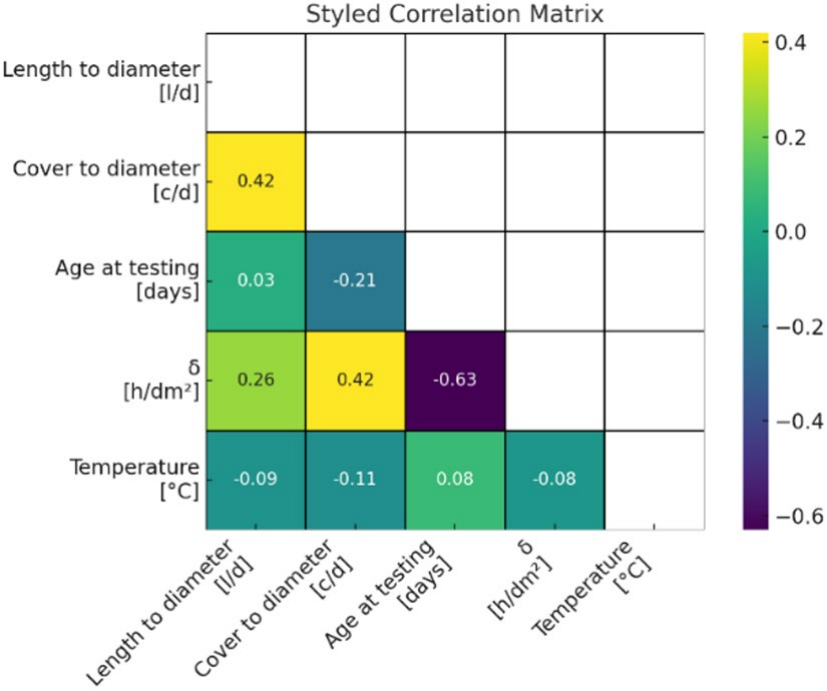
Correlation matrix of input variables.

Figure 3 offers a view of the correlation matrix for all the input variables for this study with the output variable, normalized bond strength, being omitted from the matrix to avoid self-correlation.

Analyzing the correlation analysis, it shows that no two variables of input have an overly high linear correlation which implies that the data set does not contain a high amount of multicollinearity. This is a good thing for regression analysis as it opens up the possibility for the learning algorithms to fully utilize the input parameters as it does not have to contend for dominance of a single input feature, allowing it to retrieve data from all the input parameters.

Before model training all of the variables that are continuous have been standardized to the mean value of zero for the purpose of numerical stability as well as to enable a fair comparison of the data across all the algorithms. The correlation matrix shows an overview of the linear dependencies of the input variables and also the appropriateness of the chosen variables for the intended machine learning analysis.

## Proposed methodology

This section outlines the overall computational strategy, the methods used in data preprocessing and validation, and the machine learning models used for estimating the normalized bond strength of reinforced concrete at high temperatures.

### Overall framework

**Fig. 4:**
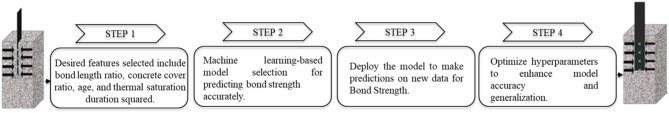
Conceptual framework of the machine learning-based bond strength prediction approach.

The overall machine learning-based framework for predicting the normalized bond strength of reinforced concrete is shown in Fig. 4. The chosen input variables, including geometry, materials, and thermal characteristics, undergo a preprocessing and standardization stage. After this, several machine learning models based on regression analysis are created using the data, and the output they predict is the normalized bond strength.

The input variables used in this analysis included the length-to-diameter ratio of the embedded bar (l/d), cover-to-diameter ratio (c/d), age at testing, thermal saturation parameter d = h/d_m^2, and exposure temperature. These parameters are used in all twelve machine learning models, and the output we try to predict is the normalized bond strength, which is defined as the ratio of the peak bond strength at elevated temperatures to the peak bond strength at ambient temperatures.

### Data preprocessing and model validation

**Fig. 5:**
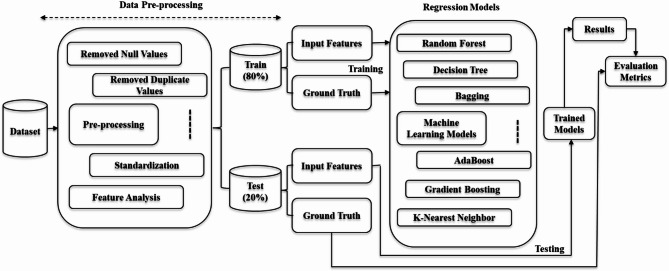
Detailed computational pipeline for data preprocessing, model training, and evaluation.

Figure 5 shows the data preprocessing steps and the framework for the computational pipeline in this study, which involves the refinement of the data, the splitting of data into training and testing sets, the repetition of cross-validation, the tuning of hyperparameters, and the evaluation of the model at its conclusion.

Before developing the models, we checked the data set for absent data and duplicate records. We removed all observations with absent data, and duplicates were not kept in the final data set. Continuous input variables were adjusted to a mean of 0 and a variance of 1 for standard numerical stability, and to facilitate the comparison of the algorithms.

To avoid data leakage, standardization parameters (mean and sd) were only calculated for the training set and then applied to the test set. The data set was randomly separated into training and testing subsets in an 80.20 ratio, with 80% of the data set to be used for model training and hyperparameter tuning, and the remaining 20% set aside for an independent evaluation of the model’s performance.

To assess model robustness and reduce overfitting, a 5-fold cross-validation repeated 10 times (i.e., 50 validation runs) was employed on the training set. The mean and standard deviation of R^2^ across folds are reported in Table 2. In each repetition, the training data were partitioned into five folds; four folds were used for training and one fold for validation, and this process was repeated such that each fold served as validation once per repetition. To further evaluate model stability and generalization capability, cross-validation results are summarized in Table 3 in terms of mean and standard deviation of R^2^ and RQME over 10 repetitions of 5-fold cross-validation. The relatively small standard deviations indicate stable performance of the main ensemble-based models across different data partitions.

**Table 3:** Cross-validation performance (mean ± standard deviation) of selected machine learning models (training set).

Model	*R*^2^ (mean ± std)	RQME (mean ± std)
XGBoost regressor	0.948 ± 0.012	6.32 ± 0.41
Gradient boosting regressor	0.944 ± 0.015	6.71 ± 0.46
Bagging regressor	0.946 ± 0.013	6.55 ± 0.44
Random trees regressor	0.942 ± 0.017	6.88 ± 0.52
Feedforward neural network (FNN)	0.945 ± 0.014	6.60 ± 0.47

### Machine learning models and hyperparameter optimization

A total of twelve regression-based machine learning algorithms were considered in this study, including Feedforward Neural Network (FNN), Random Trees, AdaBoost, Bagging, Gradient Boosting, Random Subspace Method (RSM), Radius Neighbors Regression (RNR), XGBoost, Mini-batch Gradient Descent, Polynomial Regression, Kernel Ridge Regression, and Regression Tree. These models were selected to represent a broad spectrum of learning paradigms, including linear and nonlinear regression, tree-based models, ensemble methods, instance-based learning, and neural networks. For each model, hyperparameter optimization was performed using grid search on the training set in conjunction with the repeated cross-validation framework. The primary assessment metric utilized was negative mean squared error, and each model’s hyperparameter setup was the one that resulted in a final training performance. Hyperparameter tuning was conducted via grid search with repeated cross-validation. To aid reproducibility, the main best-performing models’ hyperparmeter summaries appear in Table 4. Only the hyperparameters that significantly impacted model complexity and learning are presented.

**Table 4:** Optimal hyperparameters for selected machine learning models.

Model	Hyperparameters (optimal values)
XGBoost regressor	n_estimators = 300, max_depth = 6, learning_rate = 0.05, subsample = 0.8, colsample_bytree = 0.8
Random trees regressor	n_estimators = 200, max_features = sqrt, min_samples_leaf = 1, bootstrap = True
Gradient boosting regressor	n_estimators = 200, learning_rate = 0.10, max_depth = 3, subsample = 1.0
Feedforward neural network (FNN)	Hidden layers = [64, 32], activation = ReLU, optimizer = Adam, batch_size = 32, epochs = 200
Radius neighbors regressor	radius = 1.0, weights = distance, leaf_size = 30

#### Rationale for algorithm selection

The goal of this research was to create a systematic and exhaustive evaluation of different learning paradigms to understand how they compare to one another on typical regression problems in civil engineering. As such, the selection of various machine learning algorithms was done such that a wide scope of different methodological families was captured, and not a single class of models, such as linear and polynomial regression, tree and ensemble methods, instance-based learners, and neural networks.

To determine the value added by more sophisticated nonlinear and ensemble models, Random Tree Regression, and Polynomial Regression were included as Baseline models to set the reference point for performance. Tree-based and ensemble methods, such as Random Trees, Bagging, Gradient Boosting, AdaBoost, XGBoost, and the Random Subspace Method, were included for their demonstrated ability to address complex non-linear patterns and interactions of varying data arrays in engineering datasets.

To evaluate local vicinity-based similarity learning, instance-based model algorithms were executed via the Radius Neighbors Regressor, while the surface of feature space, which is of high dimension and is non-linearly structured, was assessed on the basis of the Ridge Regression with Kernel, whereby the technique of projection was assessed. To examine the ability of neural networks to model highly nonlinear systems, a Feedforward Neural Network was utilized to approximate the model for bond and slip relationships.

The ability to compare different constructs within this portfolio is essential. For example, one can compare simple models to advanced models, determine where frameworks lie in the parametric vs. non-parametric continuum, and analyse the differences in performance of single vs. ensemble methods. Such constructive comparisons guide one toward the more robust and stable predictors, thereby avoiding the misallocation of limited predictive power to a biased class of algorithms. Thus, from a methodological and scientific standpoint, the rationale of the current analysis derives from the presence of different, in fact, multiple, algorithmic families.

Unlike prior studies that evaluated only one or two machine learning techniques, this study performs a structured cross-family comparison including linear, tree-based, ensemble, kernel-based, instance-based, and neural network models under identical preprocessing and validation protocols. This ensures that the benchmark model is selected through systematic validation rather than algorithmic preference. Therefore, the novelty of this work lies in methodological rigor, comprehensive validation, and interpretability integration under elevated temperature conditions rather than proposing a standalone new algorithm.

### Metrics of evaluation

There are fewer than five ways of describing model performance, relative to the four that have been used in this study. Each of these four ways describes model performance differently. The four are Absolute Mean Deviation (AMD), Quadratic Mean Error (QME), Root Quadratic Mean Error (RQME), and the coefficient of determination (R^2^). These collectively address the different dimensions of prediction, including accuracy, extent, and fit.

## Experimental results and discussion

### Software and hardware environment

For the coding portion of the research, Pythion was used in Jupyter Lab together with several modules and libraries pertinent to the research. These included the fundamental libraries for the various components of data handling, visualization, and machine learning. The research is reliable, robust, and overall, of good quality due to the effective use of the machine learning and interpretability software libraries to manage the stated tasks.

The research in question made use of the Jupyter Lab in implementing the computational experiments in Python, together with the regular scientific libraries for machine learning, data visualization and processing. The evaluation and training of the model was done in a workstation with GPU acceleration. Nevertheless, the models that were reported were all done in standard desktop-class hardware. The models used in this research were not that hardware intensive, and therefore the available hardware made it easy to do quick experiments and optimization of hyperparameters.

### Performance metrics

In this case, different types of regression models available in machine learning were used and almost all models were evaluated. Each of the models were evaluated using different parameters set with more rigor. Modeling of these types serves more as a reference point in assessing the models analytically for the purpose of evaluating the model’s predictive capability.

For example, in evaluating models, the Absolute Mean Deviation (AMD) calculated is a measure that shows the average of the absolute values of the errors of the predicted values (i.e. the values of the predictions minus the actual observed values of the predictions). Better models are those with smaller values of AMD as that means the more efficient the models are. Absolute Mean Deviation (AMD) is calculated as follows:

$$\:\mathrm{A}\mathrm{M}\mathrm{D}=\frac{1}{\mathrm{n}}\sum\:_{\mathrm{i}=1}^{\mathrm{n}}\left|{\mathrm{y}}_{\mathrm{i}}-{\widehat{\mathrm{y}}}_{\mathrm{i}}\right|$$

Quadratic Mean Error (QME) is used for measuring the average squared discrepancies between anticipated and actual values. Larger errors are punished more severely than smaller errors. The computational efficiency of the model improves with decreasing QME. The following formula is used to determine Quadratic Mean Error (QME):

$$\:\mathrm{Q}\mathrm{M}\mathrm{E}=\frac{1}{\mathrm{n}}\sum\:_{\mathrm{i}=1}^{\mathrm{n}}{\left({\mathrm{y}}_{\mathrm{i}}-{\widehat{\mathrm{y}}}_{\mathrm{i}}\right)}^{2}$$

The square root of the quadratic mean error (QME) is the root quadratic mean error (RQME). With a decline in RQME, the model’s computational effectiveness increases. The formula for calculating Root Quadratic Mean Error (RQME) is as follows:

$$\:\mathrm{R}\mathrm{Q}\mathrm{M}\mathrm{E}=\:\sqrt{\mathrm{Q}\mathrm{M}\mathrm{E}}=\sqrt{\frac{1}{\mathrm{n}}\sum\:_{\mathrm{i}=1}^{\mathrm{n}}{\left({\mathrm{y}}_{\mathrm{i}}-{\widehat{\mathrm{y}}}_{\mathrm{i}}\right)}^{2}}$$

R-squared (R^2^) is a statistical metric that shows how much of the variance in the dependent variable (the goal) can be accounted for by the independent variables (the model’s characteristics). It has a value between 0 and 1, where 0 means that the model does not explain any variability and 1 means that the model fits the data perfectly and fully accounts for all variability. An improved model-data fit is indicated by a higher R^2^ score. The following formula is used to determine R-squared (R^2^) score:

$$\:{\mathrm{R}}^{2}=\:1-\:\frac{\sum\:_{\mathrm{i}=1}^{\mathrm{n}}{\left({\mathrm{y}}_{\mathrm{i}}-{\widehat{\mathrm{y}}}_{\mathrm{i}}\right)}^{2}}{\sum\:_{\mathrm{i}=1}^{\mathrm{n}}{\left({\mathrm{y}}_{\mathrm{i}}-\stackrel{-}{\mathrm{y}}\right)}^{2}}$$

where $\:n$ is the number of data points, $\:{y}_{i}$ is the actual value, $\:{\widehat{y}}_{i}$ is the predicted value, and $\bar{y}$ is the mean of the actual values.

To evaluate the models’ generalization ability and reduce overfitting, repeated 5-fold cross-validation with 10 repetitions approach was also used. The models were assessed using the chosen cross-validation approach after a grid search was carried out, with the evaluation metric set to negative mean squared error. In order to optimize the hyperparameters based on the chosen evaluation measure and the defined grid, the models were then fitted to the dataset. This method ensured a thorough evaluation of the model’s performance and parameter adjustment for best outcomes.

### Performance comparison of machine learning models

There was a total of twelve machine learning regression algorithms that were examined and classified for the study, the results of which are displayed in Table 5. Some of the models that made up the ensemble were the Feedforward Neural Network (FNN), Random Trees, AdaBoost, Bagging, Gradient Boosting Regressors, Mini-batch Gradient Descendent, Random Subspace Method (RSM), XGBoost, Polynomial Regression, Kernel Ridge Regression, Radius Neighbors Regression (RNR), and Regression Tree. The explained models offered different pros and cons, such as the versatility of ensemble techniques like Random Trees and Bagging as well as the simplicity and user friendliness of the Regression Trees.

The predictive power of the models, as the results were scrutinized by using repeated cross validation for AMD, QME, R^2^ score and RQME constituent, which were the four primary performance measurements of the output variable. Table 5 identifies the models as having excellent predictive capabilities and for having a testing AMD value range of 4.9352 to 10.1304 and a testing RQME value range of 6.8233 to 13.6275. Additionally, it is worthy of note that most of the models had a testing coefficient of determination value range of 0.8266 to 0.9417 which was consistently above 0.90.

On further careful analysis of the models, it was found that the training RQME was minimum for the XGBoost regressor model, while the testing RQME was minimum for the Radius Neighbors Regression (RNR). The training R^2^ score was maximum for the Radius Neighbors Regression (RNR), and the testing R^2^ score was maximum for the XGBoost regressor. The overall effectiveness calculated based on the above-mentioned four evaluation metrics for training and testing data of the XGBoost regressor proposed in this research was found to be the highest for addressing the primary task at hand, namely the prediction of bond strength of concrete.

**Table 5:** Performance comparison of different advanced machine learning regression models.

Regression model	Training				Testing			
	RQME	QME	AMD	*R*^2^ Score	RQME	QME	AMD	*R*^2^ Score
FNN Regressor	6.5762	43.2473	4.5330	0.9485	7.1540	51.1799	5.2077	0.9373
RandomTrees Regressor	6.8500	46.9226	4.7320	0.9454	7.4073	54.8688	5.5590	0.9259
AdaBoost Regressor	6.8886	47.4534	4.5817	0.9435	7.2095	51.9778	5.0101	0.9363
Bagging Regressor	6.4369	41.4338	4.3036	0.9506	7.1461	51.0681	4.9446	0.9374
Gradient Boosting Regressor	6.6985	44.8700	4.6741	0.9465	7.0323	49.4545	5.2382	0.9394
RSM Regressor	6.5341	42.6944	4.4558	0.9491	7.1438	51.0348	4.9352	0.9374
Radius Neighbors Regressor	6.4038	41.0095	4.0782	0.9522	6.8233	46.5574	5.1046	0.9372
XGBoost Regressor	6.1185	37.4372	3.8494	0.9517	7.8961	62.3490	5.5092	0.9417
Mini-batch GD Regressor	12.6384	159.7305	9.5447	0.8098	11.0142	121.3129	8.5363	0.8514
Polynomial Regressor	12.0304	144.7305	9.2103	0.8135	13.6275	185.7103	10.1304	0.8266
Kernel Ridge Regressor	10.2808	105.6956	7.0016	0.8770	8.2209	67.5837	5.8724	0.9088
Regression tree Regressor	6.5332	42.6832	4.2685	0.9503	7.4866	56.0494	5.4761	0.9244

After thoroughly reviewing Table 5, we see distinct trends. We could observe that apart from XGBoost regressor, which is considered to be quite effective, there are numerous alternative models that also seem to be adequat. Random Trees, Random Subspace Method, Gradient Boosting Regressor, Bagging, AdaBoost, Radius Neighbors Regression (RNR), and Feedforward Neural Network (FNN), and Regression Tree models, most of which have proven to be effective, are models that are above the average performance. This shows that the given set of challenges is effectively adaptable to most of the given machine learning regressor techniques, especially if they are mainly directed to the given area of concern, and machine learning models are proven to be a great source of options to the challenges that present the need for effective and accurate prediction.

### Feature importance and model interpretability

In practical applications of engineering, predicting bond strength and the influence of input variables is important. Even though the XGBoost regressor demonstrated the most effective performance, example feature importance analysis used a tree ensemble model and the present dataset to impart a more stable and interpretable feature ranking for input parameters related to predicted bond strength.

**Fig. 6:**
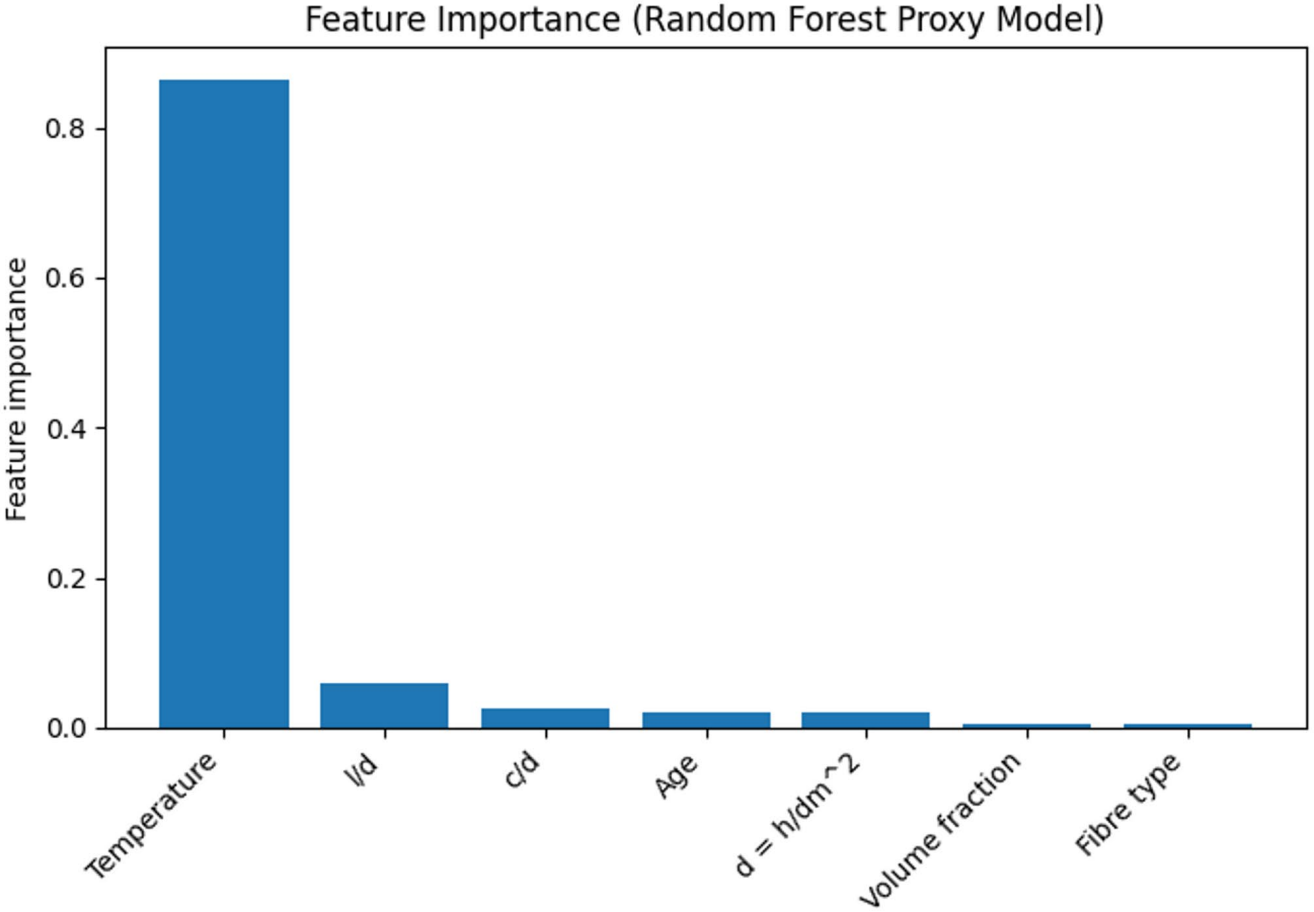
Feature importance ranking obtained from a tree-based ensemble model trained on the present dataset.

Feature importance is used within the ensemble model. Figure 6 illustrates the feature importance of the input variables. Out of all the input variables, exposure temperature was the most important variable. This is indicative of the the most important role that thermally driven bond degradation plays within the steel–concrete bond mechanism. This is aligned to the experimental studies where the bond capacity deterioration is most pronounced at higher temperatures because of microcracking and steel- concrete interface adherence loss.

The length-to-diameter ratio (l/d) and cover-to-diameter ratio (c/d) were identified as the next most important features. These geometric parameters directly control the available bond area and confinement effect, which govern stress transfer and slip behavior. A higher cover-to-diameter ratio enhances confinement and improves bond resistance, while the embedment length influences the development length and stress distribution along the bar.

The age at testing also exhibited a measurable contribution, reflecting the effect of concrete maturity and hydration on interfacial bonding. The thermal saturation parameter $\:d=h/{d}_{m}^{2}$and fibre-related variables showed moderate influence, indicating their secondary but non-negligible role in modifying bond degradation under elevated temperatures.

This interpretability analysis confirms that the machine learning framework captures physically meaningful relationships between material, geometric, and thermal parameters and the resulting bond strength, thereby increasing confidence in the applicability of the proposed approach for structural engineering assessment.

### Discussions

We analyze the findings and their potential effects and the importance and possible meaning of the analysis outcome. The differences in prediction performance levels across different machine learning algorithms is worth attention. Some of the algorithms had high levels of accuracy, meaning that they can analyze and understand the complex patterns in the prediction of the strength of bond of the concrete. Most of the studies done in this area have used XGBoost, Feed- forward Neural Network (FNN), and Artificial Neural Networks (ANN) to solve the problem of assessing bond strength of concrete. No previous studies have used this kind of dataset and problem to do a comprehensive comparative analysis of the above methods. The performance metrics such as Absolute Mean Deviation (AMD), Quadratic Mean Error (QME), Root Quadratic Mean Error (RQME) and R squared (R^2^) were analyzed.

The varying levels of prediction accuracy instigates one to find the reasons that could be used to describe the performance of the algorithm. Such a comprehensive analysis is crucial in demonstrating the specific merits and demerits of a particular algorithm used to improve the prediction of bond strength of concrete.

**Fig. 7:**
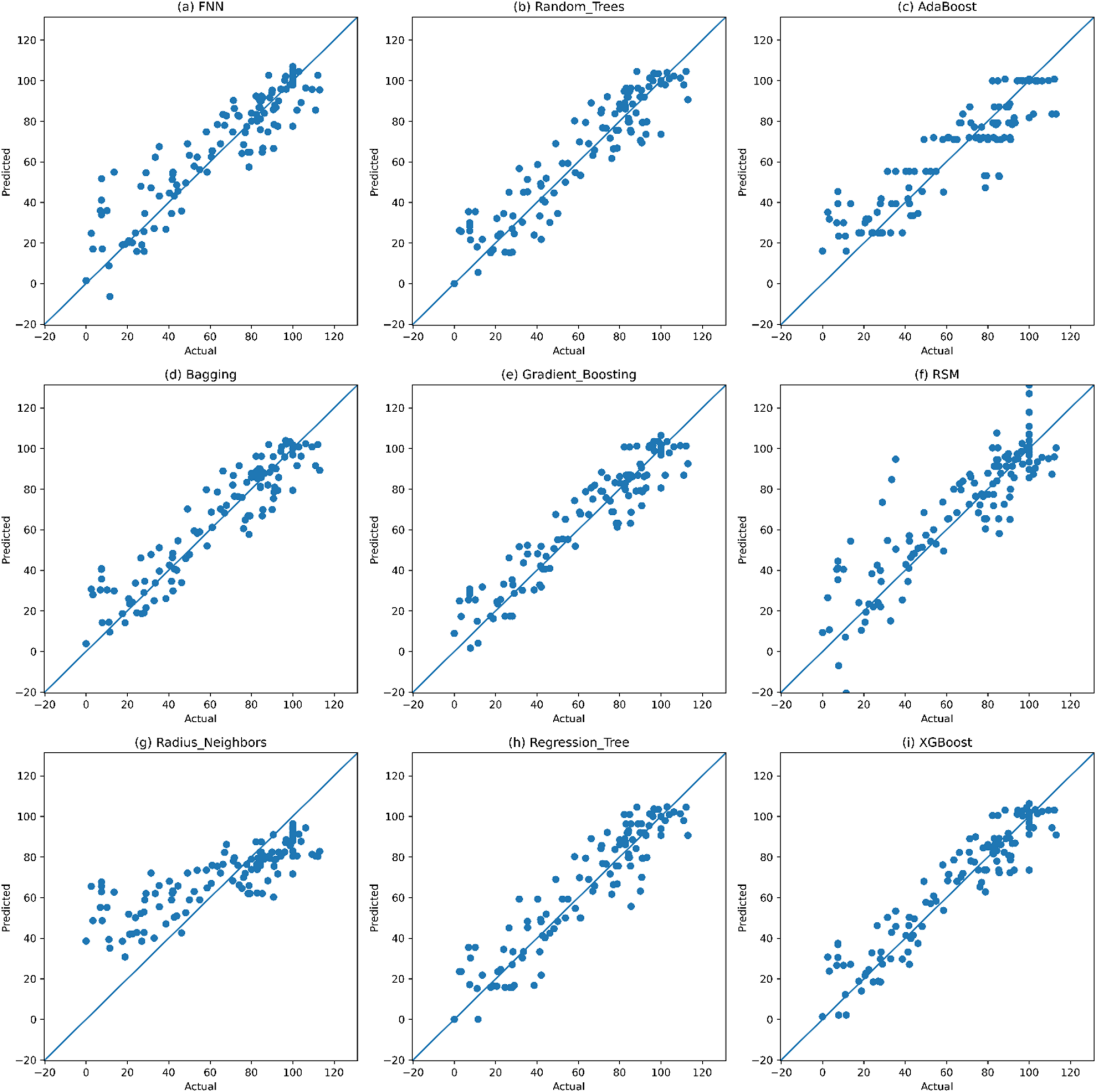
Scatter plots of best performing models.

Figure 7 shows the results of the testing R^2^ and RQME of the 9 chosen best performing regression models. Out of the twelve models of machine learning that were evaluated in the study (Table 5), the models that performed the best (9) were chosen for visualization to facilitate interpretation of comparison. The models examined in this analysis include the Feedforward Neural Network (FNN), Random Trees Regressor, AdaBoost Regressor, Bagging Regressor, Gradient Boosting Regressor, Random Subspace Method (RSM), Radius Neighbors Regressor, Regression Tree, and XGBoost Regressor. The scatter plots, however, detail the predicted bond strengths as compared to the actual bond strengths as a function of the models’ predictions which reveals the contrary strengths of the models.

The scatter plots for each algorithm and model show a varying degree of spread and clustering, as some show more clustering around the predicted values than others. The models with more data points clustered around the predicted value show more confidence in their predictions as the values predicted actual bond strengths more closely. Data points that deviate from predictions and are more spread apart decrease confidence in the predictive value of the model.

From the analyses of the scatter plots, we can learn multiple things. More specifically, we see that AdaBoost, Gradient Boosting Regressor, Bagging, and XGBoost are all at more closely packed data points and demonstrate better predictive success. Alternatively, models such as Radius Neighbors Regression (RNR), Regression Tree, Random Trees, Random Subspace Method, and Feedforward Neural Network (FNN) are more spread out and require more work. Each of the different scatter patterns gives us insight to the unique outlier clusters. Models may have difficult predicting bond strength and the outliers in the scatter plot. Locating and understanding outliers helps to improve models and better predictive success.

As noted previously, differing levels of accuracy from machine learning predictions demonstrate the relevance of the specifics of the concrete types, the environment, and the context in question. This suggests that accuracy in practical predictive modeling will require continuous improvement and tailoring of the predictive models. The machine learning models used were transparent, and there are benefits to being able to improve the accuracy of the models in order to increase the trust of concrete engineers in the models. The use of Explainable AI and Good feature selection can improve the predictive accuracy of models and increase trust in them, from a reliability perspective.

## Conclusions

This study offers a data-driven framework utilizing machine learning techniques to predict the normalized bond strength of reinforced concrete subjected to elevated temperatures. The study used a 458 data sample from investigated literature to develop and evaluate a total of 12 regression models during an extensive modeling process involving data standardization, cross-validation, and hyperparameter optimization.

Ensemble models captured bond strength degradation from high temperatures more accurately than simpler models did. The XGBoost Regressor achieved the most robust prediction of all the investigated models, with a coefficient of determination of R^2^ = 0.9417 and root mean square error of 7.9 on the testing dataset. This demonstrates competitive predictive performance compared with regression-based approaches reported in the existing literature.

Given the established mechanics of the reinforced concrete, the finding that high temperatures and the length, cover, and concrete age parameters most in combination of bond strength degradation are most indicative of the degradation of reinforced concrete provides an added dimension of interpretive and engineering value to the proposed models.

The framework developed can be seen as a useful fire safety engineering tool to provide a first order estimate of the bond strength of steel and concrete fire damaged elements. It can be used in the assessment of the fire safety of structures, evaluation of the safety of structures after a fire, durability-based design, and decision-making for the repair and strengthening of structures damaged by fire.

Perhaps the most important observation from the work completed to date is that the models developed have a number a shortcoming and these should be acknowledged. The database used for the models was developed from published Experimental Studies, and as such, each study and the results published is (sic.) subject to the variability associated with their individual testing configuration and source of materials. Within the models developed, the input parameters have been fixed, and factors such as microstructural damage, temperature and load in the heating and subsequent cooling may not be considered.

Adding new experimental data will help to enhance the database for the models developed. Future work needs to add more parameters that describe the materials used and the environment, as well as incorporating more sophisticated deep learning methodologies to model more complex nonlinear systems. In addition, integration of hybrid optimization techniques and sustainability-oriented predictive frameworks, as explored in recent studies, may further enhance model robustness and engineering applicability in future developments^43,44^. The models will be readily used by practicing engineers if the developed models are incorporated into simple software or web-based applications.

## Data Availability

Data that support the findings of this study are available from the corresponding author, [AM], upon reasonable request.

## Declarations

### Clinical trial

This study does not involve a clinical trial; therefore, registration is not applicable.
